# “Blind periods” in screening for toxoplasmosis in pregnancy in Austria – a debate

**DOI:** 10.1186/1471-2334-12-118

**Published:** 2012-05-16

**Authors:** Ulrich Sagel, Alexander Krämer, Rafael T Mikolajczyk

**Affiliations:** 1 Department of Public Health Medicine, School of Public Health, University of Bielefeld, P.O. Box 10 01 31, Bielefeld, D-33501, Germany; 2 Analyse BioLab GmbH, Eisenhandstr 4-6, Linz, A-4020, Austria; 3 Institute of Hygiene and Mikrobiology, Lower Austria State Hospital of St. Pölten-Lilienfeld, Probst-Führer-Str. 4, St. Pölten, A-3100, Austria; 4 Bremen Institute for Prevention Research and Social Medicine, Achterstr 30, Bremen, D-28359, Germany

## Abstract

Recent studies from Austria, France and Italy have shown that there is a poor adherence to the screening scheme for maternal *Toxoplasma* infections in pregnancy demonstrated by the fact that many recommended examinations are missed. This leads to undetected infections and limits our knowledge of incidence of the disease. We discuss the negative consequences of this situation on research on treatment effectiveness and the outcomes of congenital toxoplasmosis. The responsible public health institutions should assume responsibility for appropriate surveillance of the screening programme and take measures to improve screening adherence during pregnancy. Screening should start as early as possible in pregnancy and the latest test should be done at delivery. Screening schedule should allow distinguishing infections from the first, second and third trimester of pregnancy, as the risk of materno-foetal transmission and outcomes in case of foetal infections varies by time.

## Background

Few conditions in medicine are more tragic than life-long disabilities in children already acquired in pregnancy, like in congenital toxoplasmosis [1]. Some countries, most notably France and Austria, have engaged in preventive programmes to fight this disease: These countries were the first to implement population-wide, free of cost prenatal screening programmes more than three decades ago [2-4]. The idea of this approach is an early detection of maternal infection in pregnancy to implement the treatment as early as possible. Prenatal screening must be clearly distinguished from neonatal screening even if screening and treatment of newborns have beneficial effects on the course of disease [5].

Given the long-lasting experience of prenatal screening in France and Austria, there is comparably little epidemiological information from these countries about the incidence of maternal infections. Not only epidemiological research is hampered, but also the information is lacking to assess the diagnostic performance of these programmes. As stated in a recent review on the epidemiology of toxoplasmosis in pregnancy: “The prime example of minimal available data is France, …” [6]. Without doubt, this statement holds true for Austria as well. Interestingly, both countries did not complement their screening activities with appropriate surveillance systems for congenital toxoplasmosis for about three decades until at least France introduced mandatory reporting in May 2007 [2].

This situation is disappointing as the treatment concept has been questioned a few years ago. Despite earlier promising findings [4,7], more recent multi-centre observational studies failed to confirm the effectiveness of treatment provided during pregnancy [8-11]. In the context of these discussions, Denmark and Switzerland have stopped their nationwide screening programs for toxoplasmosis [12-14]. Although definitive answers on treatment effectiveness can only be obtained from randomized controlled studies [15], most recent publications provide arguments in favour of treatment effectiveness [16-19]. It must be kept in mind regarding the mentioned multi-centre studies that they cannot refute the effectiveness of treatment, as a failure to reject the null hypothesis does not mean that the alternative hypothesis must be false. More importantly, many potential biases of these studies which could disguise true effects have been discussed [20].

Why is it so difficult to determine the incidence of maternal *Toxoplasma* infections in pregnancy in a country with a long-standing screening tradition? We discuss problems encountered in the analysis of screening data from Austria.

## Discussion

We recently reported in this journal a study to determine the incidence of maternal toxoplasmosis [21]. Our analysis was based on data from the federal state of Upper Austria for the time period 2000 to 2007. Based on these experiences, we formulate some general requirements for a database from serological laboratories which would allow assessing the seroprevalence and incidence of primoinfections in pregnancy:

1. a defined catchment area

2. a defined study period (taking into consideration that pregnancy is a time period)

3. unique personal identifiers

4. age of the tested person (because of age dependency of seroprevalence!)

5. information about rural or urban residence

6. information about social status (if probability to be included into the study might depend on social factors as it can be the case for immigrants)

7. appropriate serological screening techniques

8. confirmatory testing from reference laboratory if infection is suspected by screening methods

9. standard specimen sampling scheme

10. information about gestational week and parity

11. first test as soon as possible in early pregnancy

12. latest test conducted at birth

Fortunately, the retrospectively available data from Upper Austria fulfilled many of these requirements: Using data from a single laboratory, we had access not to all but most of pregnant women of this federal state and to their personal identifiers. In contrast, there would be problems in other settings in Austria, as in many federal states several laboratories conduct screening for toxoplasmosis and data exchange is difficult due to the strict data protection regulations. Information on social factors was less important, as the study population was from all social classes. The diagnostic techniques were almost identical (single laboratory) and unchanged during the study period (indirect immunofluorescent test was replaced by another immunoassay in 2008, i.e. after the end of the study period).

A severe problem was the poor compliance with the screening scheme: Only about 30% of seronegative pregnant women had all three or more recommended tests. Apparently, this is problem is not restricted to Upper Austria: A recent study from a region in south-east France reported that only 40% of seronegative pregnant women had all seven or more screening tests according to the French scheme [22]. According to the authors, this was the first study to evaluate the compliance with the screening scheme in France. From northern Italy, adherence of less than 35% with recommended five or more screening tests was reported [23]. With regard to Austria, we are not aware of previous studies that addressed this problem.

We believe that predominantly tests in the late pregnancy were neglected in Austria: The general prenatal care programme (mother child pass, issued by Austrian Ministry of Health) schedules routine blood sampling before the 16^th^ gestational week (erythrocyte count, haemoglobin, blood group, Rhesus factor, serology for rubella immunity, *Treponema pallidum* and *Toxoplasma gondii*) and between the 25^th^ to 28^th^ gestational weeks (erythrocyte count, haemoglobin, Hepatitis B surface antigen and *Toxoplasma gondii)* if the woman was seronegative in the previous test. There is some small space to record additional blood tests in the prenatal care booklet for additional tests on toxoplasmosis, but it must be remembered by the gynaecologist if (previous tests seronegative) and when (recommendation: 8^th^ gestational month) to do so. Based on the analysis, we assume that many seronegative pregnancies had no further serological testing for *Toxoplasma* infection beyond the 28^th^ gestational week (except for some hospitals voluntarily testing at delivery, but these results are not accessible for data analysis). As our dataset did not contain information about the gestational week, we could not check this hypothesis. As a result of missing tests in the late pregnancy, large periods between the corresponding latest examinations and births remained “blind” i.e. it was not known whether infections occurred. These periods were under risk for congenital toxoplasmosis and a recent study emphasised the importance of infections in late pregnancy and of testing at delivery [24].

With long time periods without a diagnostic test (“blind periods”), it is impossible to calculate incidence from observed data only. We used two different regression models to estimate incidence and considered their limitations and biases. The estimates showed that incidence calculated from laboratory results of proven infections only suffered from severe underreporting.

In 1992, the leading Austrian toxoplasmosis experts H. Aspöck and A. Pollak presented rates of suspected infections from 1981 to 1991 referred to routine examinations from their laboratories [4]. “…, in 0.68% a primary infection during pregnancy was suspected, and in 0.32% already at the first test.” Noteworthy, about half of suspected infections were made at the first test. Usually the first test is made early in the first trimester and we would expect no more than about a third of all infections to occur in this period. Probably the problem of poor compliance with screening in the late pregnancy already existed in the 1980’s. This view is supported by the authors’ uncommented postulate [4]: “Close observation of the recommended time-table for blood sampling and the testing criteria.”

“Blind periods” at the end of pregnancy unfortunately miss those infections that are easy to diagnose, while suspected infections in the first test in pregnancy are difficult to confirm even by reference laboratories [25]. In contrast, seroconversions during pregnancy are easily determined if women were seronegative in early pregnancy. With regard to the diagnostic difficulties with suspected infections in the first test, it is important to do the first test as early as possible in pregnancy to keep the diagnostically difficult period from conception until the first test short.

“Blind periods” produce further problems: *Toxoplasma* infections are dangerous during any time in pregnancy, but the risks of transmission to the foetus and the resulting clinical affections differ from trimester to trimester [26]. Any analysis of outcomes of congenital toxoplasmosis will be limited, if some periods of pregnancy are underrepresented in assessment of infections.

Chêne and Thiébaut [27] explained the need for new treatment trials and estimated that about 260 – 350 acute infections, for example, in the second or third trimester of pregnancy have to be included per arm for a reasonable comparison of prenatal treatment with pyrimethamine-sulphadiazine to spiramycine. The authors also suggested a less demanding, placebo-controlled study, but in our view, placebo controlled study is not possible for ethical reasons. The Federal State of Upper Austria with a total population of 1.4 million inhabitants (= 1/6 of the Austrian population), could theoretically contribute about 118 – 196 cases of acute infection within an eight-year period for treatment trials, if incidence remains stable and if all infections were detected and included in the study.

In 2010, a multicentre study of 293 cases of congenital toxoplasmosis collected from six European countries investigated treatment effects on development of serious neurological sequelae [19]. The authors want their results to “be interpreted with caution because of the low number of … cases and uncertainty about the timing of maternal seroconversion.” Again, this attempt underlines the need to improve adherence to appropriate screening intervals and documentation of gestational week when screening test is conducted.

What has to be done? Austrian public health decision makers should assume responsibility for their prenatal toxoplasmosis screening program. Its performance should be assured by an appropriate epidemiological surveillance. Therefore, necessary information should be provided by prenatal care doctors, collected in electronic data sets and evaluated by public health epidemiologists. Doctors who miss recommended examinations should receive a reminder in order to improve their testing adherence.

In addition, the screening scheme should be modified to provide optimum coverage of the entire pregnancy from the very first visit of the pregnant woman in a prenatal care facility until birth. Screening intervals should at least distinguish the three trimesters of pregnancy. Keeping the gap between infection and diagnosis/treatment close, shorter intervals have been proposed [28], however economical analyses are needed to assess the cost-effectiveness of the added value of more frequent testing.

Any further research on follow-up in congenital toxoplasmosis and on efficacy of prenatal treatment would improve with complete registration of prenatal *Toxoplasma* infections given that the disease of interest is rare and that risks and outcomes of infection differ by the trimester of pregnancy.

## Summary

“Blind periods” in prenatal screening of *Toxoplasma* infections lead to missed infections and limit our knowledge of their incidence. This compromises research on treatment effectiveness and outcomes of congenital toxoplasmosis. “Blind periods” can be avoided by appropriate surveillance of the screening programme. For optimum evaluation, a minimum of electronically recorded data about the serological tests including gestational week, parity and unique personal identifiers are needed.

## Competing interests

The authors declare that they have no competing interests.

## Authors’ contributions

All authors contributed to literature search and prepared and approved the final manuscript. US prepared the first draft of the manuscript.

## Authors’ information

US is a medical microbiologist and epidemiologist and has been head of the serology unit of analyse BioLab GmbH (head: Petra Apfalter), Linz, Austria, from January 2007 to March 2009. AK is professor of public health medicine at School of Public Health, Bielefeld, Germany. RTM is clinical epidemiologist with the Bremen Institute for Prevention Research and Social Medicine.

## Pre-publication history

The pre-publication history for this paper can be accessed here:

http://www.biomedcentral.com/1471-2334/12/118/prepub
